# Editorial Notes

**Published:** 1908-03

**Authors:** 


### Editorial Notes.

**University College Colston Society.**

THE ninth annual dinner of this Society, held on January 14th, 1908, under the Presidency of Mr. George A. Wills, should in future times be looked upon as a historic occasion, for it was then that the President, in giving the toast of "The Pious Memory of Edward Colston," was able to announce that his father, Mr. Henry Overton Wills, had expressed his desire to give the munificent donation of one hundred thousand pounds towards the endowment of a University for Bristol and the West of England. This date would naturally become marked as "Founder's Day," and the speech of Sir William J. Collins, M.P., did much to emphasise the importance of the occasion. Bristol had already done good work towards the foundation of a Western University, and if anyone asked the question why there should be a University in Bristol, it was only necessary to "look at the map of England, and you will see." The sympathetic speeches of various distinguished guests, fellow-workers from Liverpool, at a meeting of the Bristol University Club on February 8th, should do much to convince the citizens of the great advantages which similar University schemes had conferred on Liverpool and other University towns in the North of England, and to prevent the recurrence of the reiterated question, Why do we want a University? and of what use will it be to us? Sir Rubert Boyce remarked that all the Universities in the north would gladly co-operate with Bristol in helping on views which were absolutely in accord with the spirit of the age. The modern University was of national importance. It was one of the lighthouses of the country, one of the national depots, and one of the national safeguards. The corporations and the Government were ready to give them every assistance, as they felt convinced that the modern University had come to stay. Chambers of Commerce gave unqualified praise to the modern University, for it helped in the every-day life to enrich commerce. Commercial men sounded the praises of Universities, and spoke of the advantage gained by having them in their midst. Professor Myers and Professor Mackay further urged their hearers to press forward with the University scheme, to go right ahead and win.

A writer on the subject of the "Multiplication of Universities" (*The Outlook*. February 1st, 1908), remarks that "the presence of a University in a manufacturing town acts as a stimulating influence to its inhabitants, and when the proper subjects are handled and particularly fostered, as a directing force to the rising generation in their industrial and commercial struggle. But it does more than this, because it also elevates the tone of the community, and teaches it to make the most of life, to seek in learning and the fruits of culture the secrets of happiness and the highest pleasures of life: to live and to work, as Goethe nobly said, 'by developing all that is in us . . . so as to make our existence satisfactory.' The citizens of Bristol, by the establishment of University College, have shown themselves to be keenly alive as regards the needs of higher education in the progressive work of their industries and their commerce. They know that knowledge, and the highest and most advanced of it, can be the only real fulcrum by which power and success can be attained." University College when started thirty years ago was liberally supported by Oxford: indeed, the Master of Balliol was one of the most ardent supporters of the scheme. He maintained that "as Mahomet cannot come to the mountain, the mountain must go to Mahomet." The multiplication of Universities has been the inevitable result. Where there were half a dozen thirty years ago there are now fifteen, and it is proposed to create more in Ireland by means of Government funds. It is too late now to raise the useless question whether there is any need for more, this has been long since settled; but here in Bristol we of all people should be the last to raise it, inasmuch as although Birmingham, Manchester, Liverpool, Leeds and Sheffield have been endowed with faculties for the giving of degrees, Bristol remains almost alone of the large cities of the country in being still without this power. If it was right that the other Universities should have been established, it must be still more right that Bristol should share with other large centres the right to have a University of its own. These arguments apply with redoubled force to the Medical Faculty, the students of which have hitherto been unable to obtain a degree without going elsewhere to find it, and hence have been obliged for the most part to rest content with the qualifications of the Medical Colleges, although their work has fully qualified them for the University medical degrees.

**The Drink Problem.** RECENT lectures given in Bristol—the one by Sir Victor Horsley and the other by Dr. Sims Woodhead—have directed the minds of the medical men and of the public to this question. These authorities are both so decided in their views that they think that nothing short of total abstinence is tenable, and that no stimulant should be given even as a medicine, excepting from a medicine bottle in carefully measured and regulated doses. The medico-sociological aspects have been fully dealt with by Dr. Kelynack in his book bearing the above title (Methuen & Co., London). Impending legislation on the subject demands that the medical profession should express its views on this two-sided question, in which so many people can discover only the one side. The dangers of excessive drinking are admitted on all hands, and even moderate drinking is to most persons unnecessary, and to some dangerous; but the medical man, from his education and training, is perhaps in a better position than most to appreciate the complexity of the problem, which is so radically and ruthlessly dealt with by the enthusiast and the faddist when they maintain that the world would be so much better if alcohol had never existed, or if it could be abolished. The uses of alcohol, both as food and as drug, are fully dealt with in the appropriate text-books, and the fallacies in the usual arguments of the rigid abstainer are not likely to divert the medical man from the moderate use of alcohol both in health and disease.

**The International Congress for Tuberculosis, at Washington, September, 1908.**

THE organisation of the English section of this Congress has been entrusted to Professors Sir Clifford Allbutt, W. Osler, and G. Sims Woodhead.

On Saturday, February 8th, a preliminary meeting was held in the large hall of the Royal Society of Medicine, when several hundred representative physicians, surgeons and pathologists were nominated to serve on a large General Committee. It was also decided to appoint an executive committee of twenty-five, six of whom should be chosen from London, nine from the provinces, five from Scotland, four from Ireland, and one from Wales.

Bristol and the West of England will probably form a local centre, and it is hoped that the district will be well represented at the Congress. Professor Walker Hall will be glad to have the names of those who intend to visit the Congress, and the titles of any proposed communications. Within a few weeks detailed information concerning the various arrangements will be available for distribution.

